# Dynamic modeling of yeast meiotic initiation

**DOI:** 10.1186/1752-0509-7-37

**Published:** 2013-05-01

**Authors:** Debjit Ray, Yongchun Su, Ping Ye

**Affiliations:** 1 School of Molecular Biosciences, Washington State University, PO Box 647520, Pullman, WA 99164, USA; 2 Biological Systems Engineering, Washington State University, Pullman, WA 99164, USA; 3 Center for Reproductive Biology, Washington State University, Pullman, WA 99164, USA

## Abstract

**Background:**

Meiosis is the sexual reproduction process common to eukaryotes. The diploid yeast *Saccharomyces cerevisiae* undergoes meiosis in sporulation medium to form four haploid spores. Initiation of the process is tightly controlled by intricate networks of positive and negative feedback loops. Intriguingly, expression of early meiotic proteins occurs within a narrow time window. Further, sporulation efficiency is strikingly different for yeast strains with distinct mutations or genetic backgrounds. To investigate signal transduction pathways that regulate transient protein expression and sporulation efficiency, we develop a mathematical model using ordinary differential equations. The model describes early meiotic events, particularly feedback mechanisms at the system level and phosphorylation of signaling molecules for regulating protein activities.

**Results:**

The mathematical model is capable of simulating the orderly and transient dynamics of meiotic proteins including Ime1, the master regulator of meiotic initiation, and Ime2, a kinase encoded by an early gene. The model is validated by quantitative sporulation phenotypes of single-gene knockouts. Thus, we can use the model to make novel predictions on the cooperation between proteins in the signaling pathway. Virtual perturbations on feedback loops suggest that both positive and negative feedback loops are required to terminate expression of early meiotic proteins. Bifurcation analyses on feedback loops indicate that multiple feedback loops are coordinated to modulate sporulation efficiency. In particular, positive auto-regulation of Ime2 produces a bistable system with a normal meiotic state and a more efficient meiotic state.

**Conclusions:**

By systematically scanning through feedback loops in the mathematical model, we demonstrate that, in yeast, the decisions to terminate protein expression and to sporulate at different efficiencies stem from feedback signals toward the master regulator Ime1 and the early meiotic protein Ime2. We argue that the architecture of meiotic initiation pathway generates a robust mechanism that assures a rapid and complete transition into meiosis. This type of systems-level regulation is a commonly used mechanism controlling developmental programs in yeast and other organisms. Our mathematical model uncovers key regulations that can be manipulated to enhance sporulation efficiency, an important first step in the development of new strategies for producing gametes with high quality and quantity.

## Background

The diploid yeast *Saccharomyces cerevisiae* undergoes mitosis in glucose medium. Upon transfer to acetate sporulation medium, cells commit to meiosis, a division process that produces four spores [1]. Meiotic initiation involves a sequential activation of signaling molecules. Importantly, expression of these molecules occurs transiently within a short time window [2-8], suggesting that protein turnover and modification are under tight regulation. These short-lived signals are important for efficient entry and successful completion of meiosis [9]. Further, interactions among these signaling molecules can lead to different levels of sporulation efficiency, as seen from yeast strains with distinct mutations or genetic background [10]. Understanding how the transient signals are generated and trigger sporulation at different efficiency represents an important first step in the development of new strategies for producing gametes with high quality and quantity.

**Figure 1:**
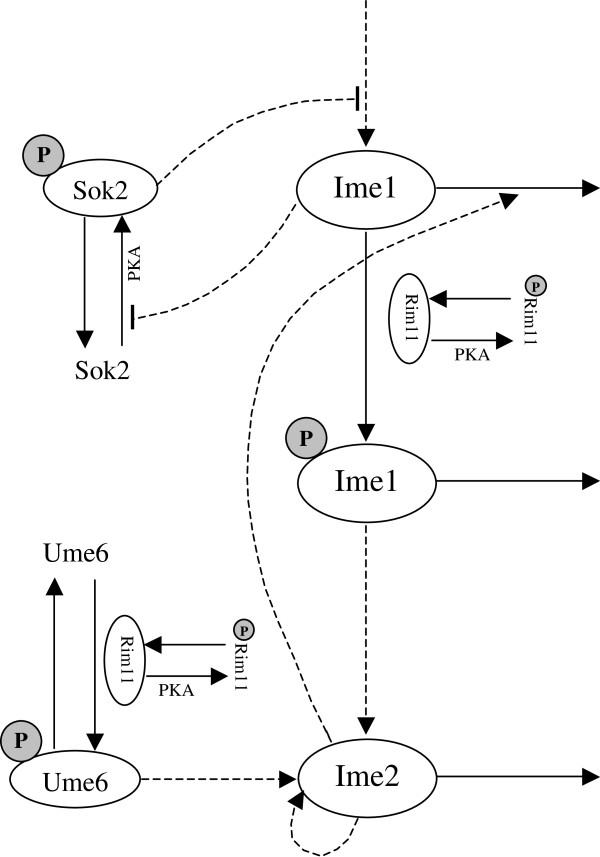
**A signaling pathway that controls yeast meiotic initiation.** Proteins enclosed in an oval are model variables. Phosphorylated proteins are labeled with the letter P in a grey circle. Solid lines represent phosphorylation, dephosphorylation, or degradation; dashed lines indicate regulatory interactions between proteins. The arrow at the end of a dashed line depicts activation; the bar at the end of a dashed line shows repression.

Many key players and their interactions that control yeast meiotic initiation have now been identified (see Figure 1) [11,12]. Ime1, the master transcriptional activator for early genes, is regulated by multiple input signals. Ime1 is repressed in the presence of glucose and activated by acetate and nitrogen depletion [13]. When glucose is present, Ime1 expression is inhibited by Sok2, which is phosphorylated by protein kinase A (PKA). Under meiotic conditions, PKA activity is reduced, resulting in dephosphorylation of Sok2 and, thereby, the release of inhibition on Ime1 [6]. Ime1 positively auto-regulates its own expression, potentially by inhibiting Sok2 activity [6]. Ime1 is also regulated by G1 cyclins (Cln3/Cdc28), which reduce *IME1* transcription and prevent Ime1 accumulation in the nucleus [14]. In contrast, transcriptional activators Msn2/4 promote *IME1* expression in the presence of acetate [15]. Similarly, Snf1, a kinase in the glucose repression pathway, stimulates expression of *IME1*[16].

Inactivation of PKA under meiotic conditions leads to enhanced activity of Rim11, a kinase that phosphorylates Ime1 and Ume6 [2,4,7]. Phosphorylation stabilizes the Ume6-Ime1 complex, which is recruited to the promoters of early meiotic genes such as *IME2* to activate their expression [17,18]. Ime2 is a kinase and functions as a positive regulator for premeiotic DNA replication and nuclear division [19]. Ime2 plays an important role in terminating expression of early meiotic genes through promoting proteolytic degradation of Ime1 [3,9]. This negative regulation ensures that Ime1 is only expressed within a narrow time window. In addition, Ime2 transcriptionally activates its own expression via upstream activation sequences [17,20]. This positive auto-regulation allows Ime2 to activate middle meiotic genes independent of Ime1. Further, mutual antagonism between Ime2 and G1 cyclins (Cln3/Cdc28) may be responsible for distinct response modes of meiotic genes to Ime1 levels [21].

*NDT80* encodes a transcription factor that activates expression of mid-meiosis genes [22]. The phosphorylated Ime1-Ume6 complex is insufficient to activate *NDT80* due to the presence of a repressor, Sum1, on its promoter. Ime2 can activate expression of *NDT80* by eliminating Sum1-mediated repression [23,24]. Ime2 further phosphorylates Ndt80, allowing Ndt80 to promote its own expression by competing with Sum1 for binding on the promoter [25]. In turn, Ndt80 is a transcriptional activator of *IME2*. Ndt80 boosts Ime2 activity during the middle stage of sporulation [26,27], and premature transcription of *NDT80* induces transcription of *IME2*[21].

Because of complex feedback regulation on the meiotic initiation pathway, mathematical modeling becomes an important tool to understand dynamic behaviors of signaling molecules and how their interactions ensure different degrees of sporulation efficiency. Feedback controls, which link the output of a circuit back to its input, are a key mechanism to stabilize cell-fate decision. Both experimental data and computational modeling suggest important roles of feedback loops in regulating mitotic entry and exit, cell growth, cell cycle, and pheromone pathways [28-33]. Negative feedback loops can generate oscillations or monotonic dynamics, while positive or double-negative feedback loops can produce bistability, i.e., having two coexisting stable steady states [34-36]. In the case of stronger positive or double-negative feedback loops, bistability can further lead to irreversibility, where a cell is locked in the post-transition state even after the stimulus disappears [29]. Likewise, feedback loops may be responsible for transient dynamics of early meiotic proteins and different sporulation outcomes.

Boolean network models—a discrete method—have been developed to simulate the dynamics of meiotic initiation pathways. One study focuses on predicting sporulation efficiency upon gene deletions, and the other explains transient transcription of *IME1* and *IME2* by introducing two hypothetical repressors to shut down gene expression [37,38]. Here, we develop an ordinary differential equation (ODE) model, a continuous method, to faithfully describe the nonlinear temporal dynamics of meiotic initiation pathways, incorporating numeric values of protein expression, kinetic rates, and time. The model depicts a signaling cascade of early meiotic proteins. Importantly, the model illustrates phosphorylation reactions and feedback loops that are crucial for directing the initiation of meiosis, based on the current knowledge available in the literature. The model correctly captures transient dynamics of meiotic proteins and accurately produces sporulation phenotypes of single-gene knockouts. We apply this model to investigate the contribution of feedback loops to transient behaviors of signaling molecules and sporulation efficiency.

## Results

### A signaling pathway that controls yeast meiotic initiation

We construct a signaling pathway to describe the initial phase of yeast meiosis based on the literature (Figure 1). The pathway includes Rim11, pUme6 (the prefix “p” stands for the phosphorylated form of the protein), pSok2, Ime1, pIme1, and Ime2. Protein synthesis, degradation, phosphorylation, and feedback regulations are depicted in the pathway (Table 1). Rim11, Ume6, and Sok2 change their phosphorylation formalism in response to external nutrients. Under meiotic conditions, reduced activity of PKA results in dephosphorylation of Rim11 and Sok2. Rim11 further mediates phosphorylation of Ume6 and Ime1. Ime1 is the master regulator of meiotic initiation. A double-negative feedback loop exists between Ime1 and pSok2. Ime1 and pUme6 together induce Ime2 expression. Subsequently, Ime2 down-regulates Ime1 through a negative feedback loop and up-regulates itself through a positive feedback loop. This signaling pathway has been converted into a set of nonlinear ordinary differential equations that describe the rate of change of proteins over time (Equations 1, 2, 3, 4, 5 and 6). The mathematical model allows for a systematic analysis of interactions between signaling molecules and how these interactions lead to different meiotic outcomes.

**Table 1:** Feedback loops in the model

**Feedback**	**Type**
Ime1 —| pSok2 —| Ime1	Double-negative feedback
Ime1 — > pIme1 — > Ime2 —| Ime1	Negative feedback
Ime2 — > Ime2	Positive feedback

The constructed model is an abstract of real pathways, incorporating major players and events. The effects of other molecules are reflected indirectly in the model. For example, we assume PKA remains constant at a low level under meiotic conditions [39,40]; the effects of upstream regulators of Ime1 (e.g., Cln3/Cdc28, Msn2/4, Snf1) are collectively represented by a general activation signal and a repression signal through pSok2; mutual inhibition between Ime2 and Cln3/Cdc28 and mutual activation between Ime2 and Ndt80 are both captured by positive auto-regulation of Ime2.

**Figure 2:**
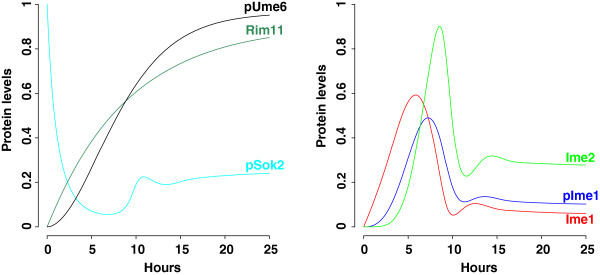
**Numerical simulations of protein levels in wild type.** The mathematical model includes Equations 1, 2, 3, 4, 5 and 6. Initial condition of all variables is 0 except for pSok2 with a value of 1. Parameter values are listed in Table 2.

Using mitotic initial conditions (all variables are 0 except pSok2 with a value of 1) and baseline parameter values (Table 2), we simulate the time-dependent dynamics of early meiotic proteins (Figure 2). The model readily generates the pattern of protein expression that is consistent with experimental evidence [2-8]. We find that early meiotic proteins are induced in a sequential and transient manner. The decline of pSok2 occurs concurrently with the rise of Rim11 and pUme6. Both unphosphorylated and phosphorylated Ime1 exhibit a transient expression peak around six hours, after which Ime2 reaches its highest expression level before decreases. At the steady state, Rim11 and pUme6 remain highly expressed, while the levels of all other proteins drop from their peak values (Table 3). In fact, the system evolves to this steady state regardless of initial conditions (Additional file 1), suggesting that a single, stable steady state exists for the ODE model. This is further confirmed by the bifurcation analysis described later (see the section “Feedback loops control sporulation efficiency”).

**Table 2:** Parameters defined in the model

**Parameter**	**Definition**	**Value**	**Reference**
Synthesis, dimension =/hour			
*s*_*ime1*_	Synthesis rate of Ime1	10	[15,43-45]
*s*_*ime2*_	Synthesis rate of Ime2	10	[39]
*s*^*’*^_*ime2*_	The maximum rate of auto-regulation-dependent Ime2 synthesis	3	Estimated
Degradation, dimension =/hour			
*d*_*ime1*_	Degradation rate of Ime1	1	[3]
*d*^*’*^_*ime1*_	The maximum rate of Ime2-activated Ime1 degradation	1	[3]
*d*_*pime1*_	Degradation rate of pIme1	1	[3]
*d*_*ime2*_	Degradation rate of Ime2	8	[3]
Phosphorylation, dimension =/hour			
*p*_*rim11*_	Phosphorylation rate of Rim11	0.01	[2], Estimated
*u*_*rim11*_	Dephosphorylation rate of Rim11	0.1	[2], Estimated
*p*_*ume6*_	Phosphorylation rate of Ume6	0.3	[18], Estimated
*u*_*ume6*_	Dephosphorylation rate of Ume6	0.01	[18], Estimated
*p*_*sok2*_	Phosphorylation rate of Sok2	0.7	[6], Estimated
*u*_*sok2*_	Dephosphorylation rate of Sok2	1	[6], Estimated
*p*_*ime1*_	Phosphorylation rate of Ime1	2	Estimated
Constant, dimensionless			
*c*_*sok2*_	Constant measuring half-maximum inhibition of Sok2 phosphorylation by Ime1	0.05	Estimated
*c*_*ime1*_	Constant measuring half-maximum inhibition of Ime1 synthesis by pSok2	0.01	Estimated
*c*_*1*_	Constant measuring half-maximum activation of Ime1 degradation by Ime2	0.01	Estimated
*c*_*2*_	Constant measuring half-maximum activation of Ime2 synthesis through auto-regulation	1.4	Estimated
*c*_*3*_	Constant measuring half-maximum activation of Ime2 degradation	2	Estimated

**Table 3:** Steady state value of variables using a mitotic initial condition and baseline parameter values

**Variable**	**Steady state value**
Rim11	0.91
pUme6	0.96
pSok2	0.25
Ime1	0.05
pIme1	0.10
Ime2	0.27

### Model validation by sporulation-deficient and proficient genes

High-throughput screens of ~4,000 yeast deletion strains have identified 267 genes required for sporulation (sporulation-deficient genes) and 102 genes whose disruption enhances sporulation efficiency (sporulation-proficient genes) [10]. Our mathematical model describes temporal dynamics of meiotic proteins encoded by five genes, among which *RIM11*, *UME6*, *IME1*, and *IME2* are sporulation-deficient genes and *SOK2* is a sporulation-proficient gene. Because the sporulation data are distinct from those used for model building, they can be applied for evaluating model performance. We virtually delete each of the five genes from the wild type model and simulate temporal dynamics of proteins in these knockout models (Additional file 2: Tables S1, S2, S3, S4 and S5, Figures S1, S2, S3, S4 and S5).

**Figure 3:**
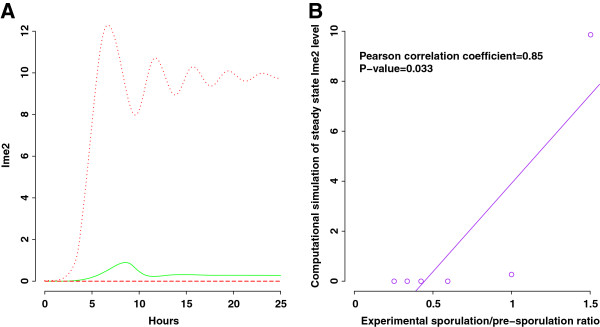
**Model validation by simulating single-gene knockouts. A**) Ime2 dynamics when deleting genes individually in the model. Red dashed line: *rim11*, *ume6*, *ime1*, and *ime2* knockouts; red dotted curve: *sok2* knockout; green curve: wild type. Mathematical models of single-gene knockouts are described in Additional file 2: Tables S1, S2, S3, S4 and S5. **B**) Pearson correlation between experimental sporulation/pre-sporulation ratios and simulated Ime2 values at steady state for five single-gene knockouts and wild type.

Ime2 is used as the model readout for sporulation phenotypes because it is the most downstream protein that reflects changes in all others in the pathway. We find that Ime2 levels remain at zero in the knockout models of sporulation-deficient genes *RIM11*, *UME6*, *IME1*, and *IME2* (Figure 3A). Virtual deletion of *IME2* results in non-transient expression of Ime1 (Additional file 2: Figure S5), consistent with previous experimental observation [3]. In contrast, for the knockout model of *SOK2*, a sporulation-proficient gene, Ime2 exhibits damped oscillations and enhanced expression compared to the wild type model (Figure 3A). To further quantitatively evaluate the ODE model for sporulation phenotypes, we calculate the Pearson correlation coefficient between experimentally determined sporulation/pre-sporulation ratios and simulated steady state levels of Ime2 for five gene knockouts and wild type (Figure 3B). Significant correlation is observed between the measured and predicted sporulation efficiency (Pearson correlation = 0.85, P-value = 0.033). These results suggest that the mathematical model correctly captures sporulation phenotypes of single-gene deletions. Once the model is validated, we can then use the model to explain and predict the role of feedback loops in regulating sporulation efficiency and transient behaviors of signaling molecules. Noteworthily, the constructed model (Figure 1) may be one of many models that could generate protein dynamics and knockout phenotypes in agreement with experimental evidences. The identification of other potential models requires scanning all possible topologies linking the six proteins that can satisfy the current knowledge of yeast meiosis.

### A global analysis of parameter sensitivity

**Figure 4:**
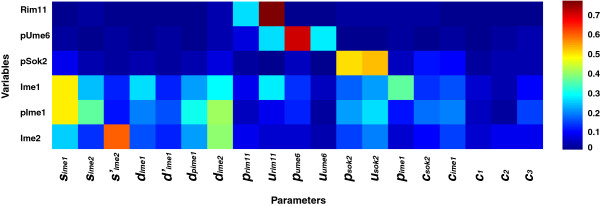
**Analyses of global parameter sensitivity.** Global effects of parameters on model variables are investigated by simultaneously varying all 19 parameters within a range of one order of magnitude larger and smaller than baseline values. A random sample of each parameter is generated from the range with a uniform distribution; the sampling is performed 5,000 times to calculate parameter sensitivities. Sensitivity value is between 0 and 1; the larger the value, the more important a parameter is to the output of a variable.

When *in silico* models include a large number of parameters describing biological processes, it is critical to understand the role of each parameter in variations of model outcome. Sensitivity analysis is used to investigate which parameters have the greatest influence on model output. It can help identify key parameters—and, thus, the associated biological processes—that determine distinct outcomes. We perform multi-parametric sensitivity analysis on the ODE model [41]. The response of six variables is examined by simultaneously varying all 19 parameters in the model over a wide range of choices. Sensitivity is in the range of 0 and 1; more important parameters are associated with larger sensitivity values (Figure 4).

The overall pattern indicates that early meiotic proteins are sensitive to parameters that directly regulate their homeostasis. Levels of Rim11, pUme6, and pSok2 are mainly affected by phosphorylation and dephosphorylation. Rim11 and pSok2 are more sensitive to dephosphorylation than phosphorylation (*u*_*rim11*_, *u*_*sok2*_), but the opposite is true for pUme6 (*p*_*ume6*_). The findings are consistent with the active forms of these proteins in meiosis. Synthesis and phosphorylation are most important to alter the Ime1 level (*s*_*ime1*_, *p*_*ime1*_); both processes directly determine the gain or loss of Ime1. The level of pIme1 is primarily modulated by the synthesis of Ime1 and degradation of Ime2 (*s*_*ime1*_, *d*_*ime2*_). Ime1 synthesis indirectly controls pIme1 through regulating Ime1; Ime2 degradation indirectly influences pIme1 through Ime2-activated Ime1 degradation. Parameters that control Ime2 auto-regulation and degradation have the greatest influence on Ime2 variations (*s*^*’*^_*ime2*_, *d*_*ime2*_).

### Feedback loops control transient expression of signaling molecules

Feedback regulations are important for coordinated and transient behaviors of developmental systems [33,37]. Ime1 and Ime2 exhibit orderly and transient expression during meiosis (Figure 2). These short-lived signals are critical for the successful completion of sporulation [9]. We investigate how feedback loops affect dynamics of early meiotic proteins. A total of three feedback loops are described in the model: double-negative feedback between pSok2 and Ime1, negative feedback from Ime2 to Ime1, and auto-positive feedback of Ime2 (Table 1). We up-regulate, down-regulate, or delete each feedback loop through manipulating corresponding parameters. Protein dynamics are monitored in these *in silico* perturbation experiments.

#### ***Double-negative feedback between pSok2 and Ime1***

**Figure 5:**
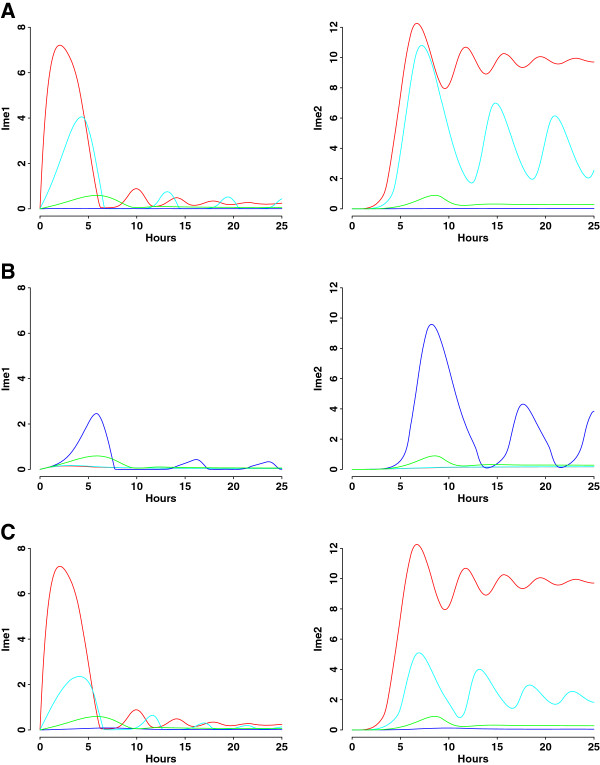
**Simulation analyses of double-negative feedback loop between pSok2 and Ime1. A**) Ime1 and Ime2 dynamics when varying *c*_*ime1*_, the constant measuring half-maximum inhibition of Ime1 synthesis by pSok2. Red curve: *c*_*ime1*_ = ∞ (deleting the inhibition); cyan curve: *c*_*ime1*_ = 0.1 (decreasing the inhibition); blue curve: *c*_*ime1*_ = 0.001 (increasing the inhibition); green curve: *c*_*ime1*_ = 0.01 (baseline value). **B**) Ime1 and Ime2 dynamics when varying *c*_*sok2*_, the constant measuring half-maximum inhibition of Sok2 phosphorylation by Ime1. Red curve: *c*_*sok2*_ = ∞ (deleting the inhibition); cyan curve: *c*_*sok2*_ = 0.5 (decreasing the inhibition); blue curve: *c*_*sok2*_ = 0.005 (increasing the inhibition); green curve: *c*_*sok2*_ = 0.05 (baseline value). **C)** Ime1 and Ime2 dynamics when varying *c*_*ime1*_ and *c*_*sok2*_ simultaneously. Red curve: *c*_*ime1*_ = ∞ and *c*_*sok2*_ = ∞ (deleting the feedback loop); cyan curve: *c*_*ime1*_ = 0.1 and *c*_*sok2*_ = 0.5 (decreasing the feedback loop); blue curve: *c*_*ime1*_ = 0.001 and *c*_*sok2*_ = 0.005 (increasing the feedback loop); green curve: *c*_*ime1*_ = 0.01 and *c*_*sok2*_ = 0.05 (baseline value). The mathematical model of the feedback knockout is described in Additional file 2: Table S6.

Phosphorylated Sok2 is an upstream repressor of Ime1, and, conversely, Ime1 inhibits Sok2 phosphorylation, forming a double-negative feedback loop. We first evaluate the effect of inhibition from pSok2 to Ime1, by varying *c*_*ime1*_, the constant measuring half-maximum inhibition of Ime1 synthesis by pSok2 (Figure 5A). When this inhibition is enhanced (blue curves), no expression is observed for Ime1 and Ime2; the entire meiotic pathway is turned off. When this inhibition is partially or completely relieved (cyan and red curves), damped oscillations appear for both Ime1 and Ime2, and Ime2 reaches a higher steady state than wild type. Next, we evaluate the inhibition from Ime1 to pSok2, by varying *c*_*sok2*_, the constant measuring half-maximum inhibition of Sok2 phosphorylation by Ime1 (Figure 5B). Manipulating the inhibition toward pSok2 produces the opposite effect: increasing the inhibition (blue curves) activates the meiotic pathway, while decreasing or dismissing the inhibition (cyan and red curves) represses the pathway. We further investigate the feedback loop by simultaneously varying *c*_*ime1*_ and *c*_*sok2*_ (Figure 5C). The results are similar to those from manipulating the inhibition from pSok2 to Ime1. In particular, the feedback knockout results in enhanced Ime2 expression (Additional file 2: Table S6, Figure S6). The orderly and transient behavior of both Ime1 and Ime2, however, are not affected by manipulating the different arm of feedback loop or the entire feedback loop.

#### ***Negative feedback from Ime2 to Ime1***

**Figure 6:**
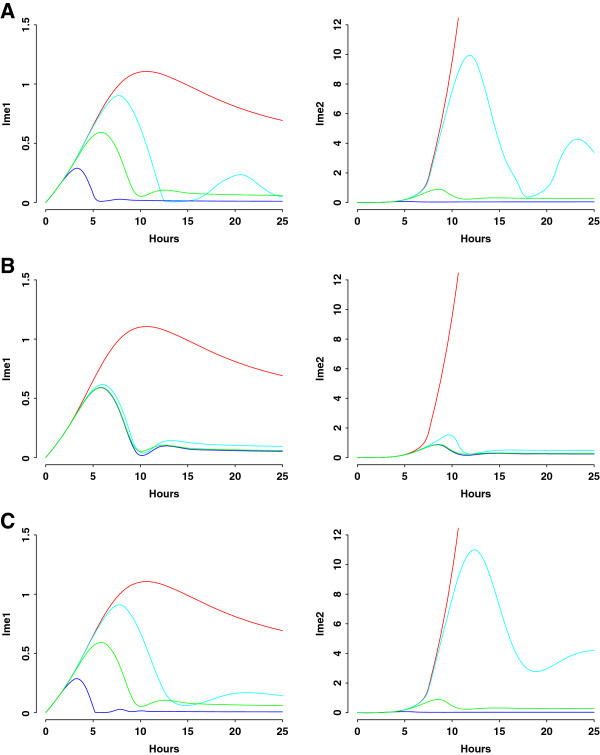
**Simulation analyses of negative feedback loop from Ime2 to Ime1. A**) Ime1 and Ime2 dynamics when varying *d’*_*ime1*_, the maximum rate of Ime2-activated Ime1 degradation. Red curve: *d’*_*ime1*_ = 0 (deleting the feedback loop); cyan curve: *d’*_*ime1*_ = 0.1 (decreasing the feedback loop); blue curve: *d’*_*ime1*_ = 10 (increasing the feedback loop); green curve: *d’*_*ime1*_ = 1 (baseline value). **B**) Ime1 and Ime2 dynamics when varying *c*_*1*_, the constant measuring half-maximum activation of Ime1 degradation by Ime2. Red curve: *c*_*1*_ = ∞ (deleting the feedback loop); cyan curve: *c*_*1*_ = 0.1 (decreasing the feedback loop); blue curve: *c*_*1*_ = 0.001 (increasing the feedback loop); green curve: *c*_*1*_ = 0.01 (baseline value). **C**) Ime1 and Ime2 dynamics when varying *d’*_*ime1*_ and *c*_*1*_ simultaneously. Red curve: *d’*_*ime1*_ = 0 and *c*_*1*_ = ∞ (deleting the feedback loop); cyan curve: *d’*_*ime1*_ = 0.1 and *c*_*1*_ = 0.1 (decreasing the feedback loop); blue curve: *d’*_*ime1*_ = 10 and *c*_*1*_ = 0.001 (increasing the feedback loop); green curve: *d’*_*ime1*_ = 1 and *c*_*1*_ = 0.01 (baseline value). The mathematical model of the feedback knockout is described in Additional file 2: Table S7.

Protein destruction is a commonly used mechanism controlling cell cycle transitions [28]. Ime1 activates Ime2, while Ime2 inhibits Ime1 by promoting its degradation. Previous studies indicate that negative feedback loops are required for transient transcription of *IME1*[3,9,37]. We test whether negative feedback from Ime2 to Ime1 is responsible for confining expression of early meiotic proteins to a narrow window. When increasing *d’*_*ime1*_, the maximum rate of Ime2-activated Ime1 degradation, the negative feedback is enhanced (blue curve). Both amplitude and duration of Ime1 peak decrease, which lead to no expression of Ime2 (Figure 6A). When the feedback is reduced or dismissed by changing *d’*_*ime1*_ (cyan and red curves), we observe not only increased peak height but also increased peak width for both Ime1 and Ime2. Ime2 rises to infinity in the feedback knockout model (Figure 6A, Additional file 2: Table S7, Figure S7). The negative feedback can also be manipulated by varying *c*_*1*_, the constant measuring half-maximum activation of Ime1 degradation by Ime2 (Figure 6B), or by varying *d’*_*ime1*_ and *c*_*1*_ simultaneously (Figure 6C). The results are similar to those of changing *d’*_*ime1*_, suggesting that the negative feedback ensures transient expression of both Ime1 and Ime2.

#### ***Auto-positive feedback of Ime2***

**Figure 7:**
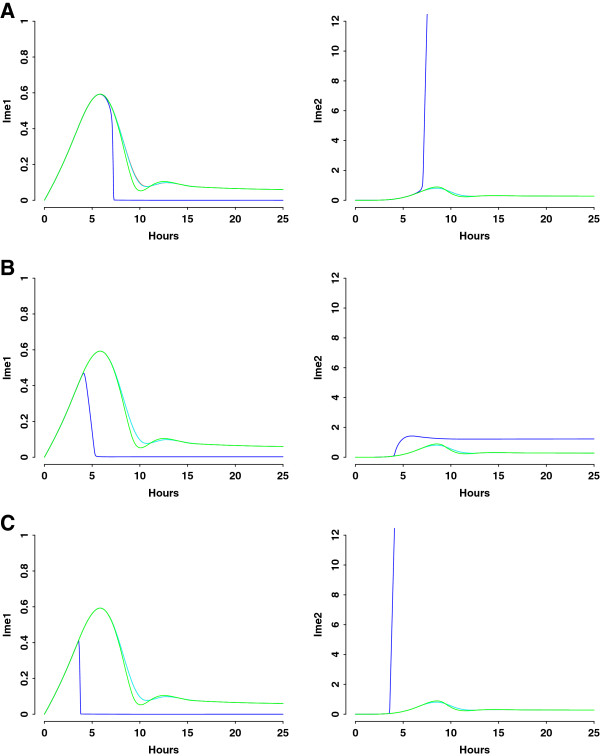
**Simulation analyses of positive feedback loop of Ime2. A**) Ime1 and Ime2 dynamics when varying *s’*_*ime2*_, the maximum rate of auto-regulation-dependent Ime2 synthesis. Red curve: *s’*_*ime2*_ = 0 (deleting the feedback loop); cyan curve: *s’*_*ime2*_ = 0.3 (decreasing the feedback loop); blue curve: *s’*_*ime2*_ = 30 (increasing the feedback loop); green curve: *s’*_*ime2*_ = 3 (baseline value). **B**) Ime1 and Ime2 dynamics when varying *c*_*2*_, the constant measuring half- maximum activation of Ime2 synthesis through auto-regulation. Red curve: *c*_*2*_ = ∞ (deleting the feedback loop); cyan curve: *c*_*2*_ = 14 (decreasing the feedback loop); blue curve: *c*_*2*_ = 0.14 (increasing the feedback loop); green curve: *c*_*2*_ = 1.4 (baseline value). **C**) Ime1 and Ime2 dynamics when varying *s’*_*ime2*_ and *c*_*2*_ simultaneously. Red curve: *s’*_*ime2*_ = 0 and *c*_*2*_ = ∞ (deleting the feedback loop); cyan curve: *s’*_*ime2*_ = 0.3 and *c*_*2*_ = 14 (decreasing the feedback loop); blue curve: *s’*_*ime2*_ = 30 and *c*_*2*_ = 0.14 (increasing the feedback loop); green curve: *s’*_*ime2*_ = 3 and *c*_*2*_ = 1.4 (baseline value). The mathematical model of the feedback knockout is described in Additional file 2: Table S8.

Multiple lines of evidence support positive auto-regulation of Ime2: transcriptional activation of its own expression [17,20], mutual antagonism between Ime2 and G1 cyclins (Cln3/Cdc28) [21], and mutual activation between Ime2 and Ndt80 [21,23-27]. To examine the role of auto-regulation in protein dynamics, we vary *s’*_*ime2*_, the maximum rate of auto-regulation-dependent Ime2 synthesis (Figure 7A), *c*_*2*_, the constant measuring half-maximum activation of Ime2 synthesis through auto-regulation (Figure 7B), or both (Figure 7C). Up-regulation of the feedback (blue curve) causes earlier decline of Ime1 peak and earlier increase of Ime2 to a higher level than wild type. The abrupt drop of Ime1 is due to negative feedback from enhanced expression of Ime2. Ime2 is more sensitive to variations in *s’*_*ime2*_ than in *c*_*2*_, consistent with the global analysis of parameter sensitivity (Figure 4). When auto-positive feedback is down-regulated or deleted (cyan and red curves), both Ime1 and Ime2 exhibit similar dynamics as for wild type (Additional file 2: Table S8, Figure S8). These results indicate that the auto-regulation is responsible for transient Ime2 expression. The transient dynamics of Ime1, however, are preserved regardless of the strength of auto-regulation.

### Feedback loops control sporulation efficiency

Feedback regulations are known to control cell fate decision [34]. In the context of yeast meiosis, feedback loops linking early proteins may be responsible for distinct sporulation efficiencies, traced by steady state levels of Ime2, the most downstream protein in the model. We perform bifurcation analyses on parameters governing feedback loops to determine which ones cause changes in the stability of Ime2 equilibrium.

#### ***Double-negative feedback between pSok2 and Ime1***

**Figure 8:**
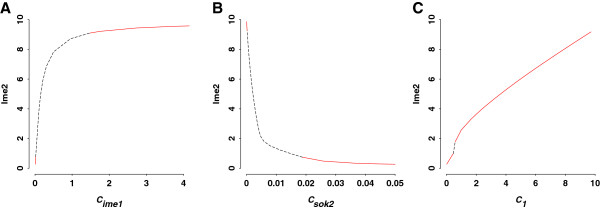
**Bifurcation analyses of double-negative feedback loop between pSok2 and Ime1 and negative feedback loop from Ime2 to Ime1. A**) Steady state value of Ime2 as a function of *c*_*ime1*_, the constant measuring half-maximum inhibition of Ime1 synthesis by pSok2. **B**) Steady state value of Ime2 as a function of *c*_*sok2*_, the constant measuring half-maximum inhibition of Sok2 phosphorylation by Ime1. **C**) Steady state value of Ime2 as a function of *c*_*1*_, the constant measuring half-maximum activation of Ime1 degradation by Ime2. Red segments represent stable steady states, whereas black segments trace unstable steady states.

Mutual antagonism between pSok2 and Ime1 is controlled by *c*_*ime1*_ and *c*_*sok2*_, half-maximum inhibition constants. Varying either *c*_*ime1*_ or *c*_*sok2*_ produces two stable steady states separated by an unstable steady state (Figure 8A-B). When both parameters are close to their baseline values (*c*_*ime1*_ = 0.01, *c*_*sok2*_ = 0.05), the default equilibrium value of Ime2 is obtained (Ime2 = 0.27). When *c*_*ime1*_ increases or *c*_*sok2*_ decreases, implying that Ime1 wins over pSok2, Ime2 reaches a higher stable state. This higher state indicates that sporulation efficiency can be improved by manipulating double-negative feedback loop between pSok2 and Ime1.

PKA mediates phosphorylation of Sok2 and Rim11. To examine whether PKA is also a bifurcation parameter, we vary the phosphorylation rates of Sok2 and Rim11 simultaneously (Additional file 2: Figure S9). Two stable steady states, separated by an unstable steady state, are again observed. One stable state is the default equilibrium value of Ime2. When PKA activity is reduced, represented by lowering phosphorylation rates, the second stable state of higher Ime2 appears, corresponding to elevated sporulation efficiency. This result suggests that sporulation efficiency can also be improved by suppressing PKA activity.

#### ***Negative feedback from Ime2 to Ime1***

Parameter *c*_*1*_ is the half-maximum constant of Ime2 inhibition on Ime1. Changing *c*_*1*_ results in two stable steady states, separated by a very short segment of unstable steady state (Figure 8C). Baseline value (*c*_*1*_ = 0.01) leads to the default Ime2 equilibrium (Ime2 = 0.27). The inhibition from Ime2 to Ime1 decreases with the increase of *c*_*1*_, producing enhanced Ime2 level. This indicates that sporulation efficiency can be improved by repressing negative feedback from Ime2 to Ime1.

#### ***Auto-positive feedback of Ime2***

**Figure 9:**
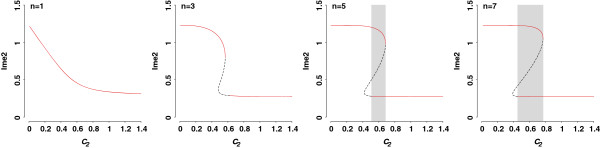
**Bifurcation analyses of positive feedback loop of Ime2.** Steady state value of Ime2 as a function of *c*_*2*_, the constant measuring half-maximum activation of Ime2 synthesis through auto-regulation. The use of different Hill coefficients, *n*, as indicated on the plot reveals monostability and bistability. Red segments represent stable steady states, whereas black segments trace unstable steady states. The bistability region is indicated by grey.

Auto-regulation of Ime2 is approximated by a Hill function with the coefficient of 5. We use high Hill coefficient to define cooperative and ultrasensitive regulatory processes because this feedback loop represents not only auto-regulation but also multiple interactions between Ime2 and other molecules (e.g., Cln3/Cdc28, Ndt80). Parameter *c*_*2*_ is the half-maximum constant of Ime2 auto-regulation. Plotting Ime2 as a function of *c*_*2*_ (Figure 9) shows the default Ime2 equilibrium (Ime2 = 0.27) at baseline parameter value (*c*_*2*_ = 1.4). When *c*_*2*_ is less than 0.5, the auto-regulation is enhanced, which leads to a higher steady state response of Ime2. With *c*_*2*_ in the region of 0.5-0.7, the system becomes bistable. Ime2 can take two different values, characterizing states of default and higher sporulation efficiency. These two stable states can be reached for the same set of parameters depending on initial conditions.

The Hill coefficient determines the switch-like behavior of Ime2 equilibrium. We find that the range of *c*_*2*_ in which the system exhibits bistability is sensitive to the Hill coefficient (Figure 9). The system is monostable with coefficients of 1 or 3, since one value of *c*_*2*_ corresponds to a single value of Ime2. Higher coefficients result in the transition from a monostable to a bistable system. A Hill coefficient of 7 expands the region of bistability across a broad range of parameter space, making the cell fate more robust with respect to perturbations in the feedback loop. This result indicates that cooperativity of Ime2 molecules is essential for producing bistable sporulation outcomes.

## Discussion

Precise regulation of a gene cascade in a coordinated manner is required for initiating a developmental program at the right time. This is often achieved through the activation of an upstream master regulator, which is controlled by multiple input signals and further regulates expression of downstream genes. Downstream genes, in turn, feed back to the regulator to modulate the entire pathway activity. The combinational nature of feedback loops ensures correct temporal dynamics of a developmental program [34-36].

The goal of this study is to understand and predict the effect of the control structure, i.e., feedback loops, on transient expression of early meiotic proteins and on distinct sporulation efficiencies observed in budding yeast. We construct a meiotic initiation pathway using an ODE-based model that includes regulation of Ime1, the master regulator, and five other early-meiotic proteins. We consider three feedback loops that control expression of these proteins: double-negative feedback between pSok2 and Ime1, negative feedback from Ime2 to Ime1, and auto-positive feedback of Ime2. In particular, Ime1 is controlled by an upstream inhibitor, Sok2, and a downstream inhibitor, Ime2.

The model is capable of simulating orderly and transient expression of meiotic proteins, without relying on putative repressors to shut down gene expression [37]. The model is further validated by quantitative sporulation phenotypes of single-gene knockouts. We analyze the sensitivity of the model and find that proteins are sensitive to processes that directly regulate their levels. Subsequently, we perform *in silico* experiments on the model to understand the feedback mechanism on controlling transient protein expression and different sporulation efficiencies. The strength of mathematical models is that they serve as easily manipulatable systems for many perturbation experiments that are either extremely difficult or not tractable in a wet-lab setting.

The new insights gained from this study are two fold. First, we conclude that feedback loops play important roles in terminating expression of early meiotic proteins. Negative feedback from Ime2 to Ime1 is responsible for transient expression of both Ime1 and Ime2, in agreement with previous finding [3,9,37]. However, our study elucidates, for the first time, that the auto-positive feedback of Ime2 also ensures that Ime2 expression is confined to a narrow window. In our model, Ime2 responds in a graded mode to the Ime1 levels (Figures 5, 6 and 7), consistent with experimental observation that transcription of early meiotic genes is regulated by a gradient effect produced by Ime1 [21].

More importantly, the second new insight from exploration of the model is that feedback loops are responsible for tuning the efficiency of meiotic pathways. We perform bifurcation analyses on feedback loops using the equilibrium value of Ime2 as the pathway readout. We find that, by adjusting each of the two arms of mutual inhibition between pSok2 and Ime1, the system is able to move from a default meiotic state to a more efficient meiotic state. Similarly, by manipulating the strength of negative feedback loop from Ime2 to Ime1, the model readily produces a default meiotic state and a more efficient meiotic state. Auto-positive regulation of Ime2 is characterized by the Hill function with a high coefficient, providing a simple, reasonably accurate approximation for multiple regulations occurring on Ime2. This positive feedback generates a bistable pathway with two alternative stable steady states—the default meiotic state and a more efficient meiotic state. The robustness of bistability is sensitive to the Hill coefficient, indicating a strong cooperativity and nonlinearity in the response of Ime2 to the feedback. We propose that the combinational feedback regulation controls sporulation efficiency and guarantees that meiotic initiation proceeds in an accurate temporal scale.

Our mathematical model constitutes physical interactions of early meiotic proteins and provides mechanistic insights into ordered appearance of key regulators and sporulation efficiency. Such a model illustrates how different feedback regulations are integrated in the signaling pathway to cause changes in protein expression and meiotic outcome. The model is a reduced system of differential equations, including only Rim11, Ume6, Sok2, Ime1, and Ime2. Other proteins and/or links involved in meiotic initiation are traced indirectly. Validation using deletion mutants of meiotic genes suggests that major regulatory interactions have been captured. We demonstrate that the ordinary differential equation method can depict the most prominent features of signaling pathway during yeast meiotic initiation. Our mathematical model allows for uncovering key regulations that can lead to manipulation of the pathway to enhance sporulation efficiency. This represents an important first step in designing new strategies for producing gametes with high quality and quantity.

## Conclusions

We develop a dynamic model to describe signaling pathways that operate during yeast meiotic initiation. Our study suggests that both positive and negative feedback loops control transient expression of early meiotic proteins, and multiple feedback loops regulate the efficiency of meiotic progression. Thus, yeast meiotic initiation is the consequence of systems-level feedback that leads cells into distinct sporulation states.

## Methods

### An ODE model

We formulate a mathematical model to describe the temporal dynamics of a signaling pathway that controls yeast meiotic initiation. The kinetics is based on SK1, a strain commonly used for studying yeast meiosis. Six proteins in either phosphorylated or unphosphorylated form are model variables. All variables are dimensionless and represent relative protein levels within the range of zero to one unit. The rate of change of protein levels is captured by ordinary differential equations, which include terms describing protein synthesis, degradation, phosphorylation, dephosphorylation, as well as regulatory activation and repression.

(1) $$\frac{\text{dRim}11}{\mathrm{dt}}=u_{\text{rim}11}\cdot \left(1-\text{Rim}11\right)-p_{\text{rim}11}\cdot \text{Rim}11$$

(2) $$\frac{\text{dpUme}6}{\mathrm{dt}}=p_{\text{ume}6}\cdot \text{Rim}11\cdot \left(1-\text{pUme}6\right)-u_{\text{ume}6}\cdot \text{pUme}6$$

(3) dpSok2dt=psok2·csok2csok2+Ime1·1−pSok2−usok2·pSok2

(4) dIme1dt=sime1·cime1cime1+pSok2−pime1·Rim11·Ime1−dime1·Ime1−dime1,·Ime2·Ime1c1+Ime1

(5) dpIme1dt=pime1·Rim11·Ime1−dpime1·pIme1

(6) dIme2dt=sime2·pUme6·pIme1+sime2,·Ime25c25+Ime25−dime2·Ime2c3+Ime2

Phosphorylated or unphosphorylated Rim11, Ume6, and Sok2 are variables in Equations 1, 2 and 3. Because these three proteins exhibit uniform expression levels over the entire course of sporulation [2,39,40,42], the total amount of phosphorylated and unphosphorylated forms of each protein is assumed to be constant (one unit). Phosphorylation and dephosphorylation are the only events described. PKA catalyzes phosphorylation of Rim11 and Sok2, although it is not included explicitly as a variable in the model. Phosphorylation of Sok2 is inhibited by Ime1, which is described using an inhibitory Hill function [6]. Phosphorylation of Ume6 is mediated by Rim11 and modeled by mass action [4,7].

Unphosphorylated and phosphorylated Ime1 are variables in Equations 4 and 5. Ime1 synthesis is inhibited by Sok2, as described using an inhibitory Hill function [6]. Ime1 has a basal degradation rate, plus an Ime2-induced degradation [3,9]. We assume that the negative feedback-dependent rate of Ime1 degradation is proportional to a Hill function. Phosphorylation of Ime1 is mediated by Rim11 and defined by mass action [2].

Equation 6 describes the rate of change of Ime2, the most downstream protein in the model. The synthesis of Ime2 depends on phosphorylated forms of Ime1 and Ume6 [17,18]. Ime2 synthesis is further enhanced through positive auto-regulation [17,20]. We assume that the auto-activation obeys cooperative kinetics, modeled by a Hill function with the coefficient of 5. Ime2 degradation occurs in a density-dependent manner.

The ODE model in the Systems Biology Markup Language format is provided as Additional file 3. The model is also available at the BioModels database (http://www.ebi.ac.uk/biomodels/, MODEL1303060000).

### Initial conditions of variables

We consider a mitotic initial state. All variables except for pSok2 are set to zero because a very low level of meiosis-specific proteins could be detected during vegetative growth [3,7,8,39,40,42]. Sok2 functions as a positive regulator of mitosis but as a negative regulator of meiosis [6,12]. Thus, pSok2 is given the maximum level of one as the initial condition.

### Parameter values

Parameters are either rate coefficients with a dimension of per hour or dimensionless constants (Table 2). Parameter values are estimated from the literature when they are available. When no data exist for parameters, values are manually explored over several orders of magnitude. Baseline values of parameters are determined by comparing model-generated output with experimental results in the literature [2-8,25] and by constraining variable values in the range of zero and one. Outlined below is how we estimate parameter values.

Synthesis rates of genome-wide proteins have been calculated from experiments when yeast cells grow mitotically in glucose medium [43]. Fold increase in the *IME1* promoter activity has been measured when cells are transferred from glucose to sporulation medium [15,44,45]. Synthesis rate of Ime1 is estimated to be 10/hour during meiosis based on the above two measurements. Protein synthesis of sporulating cells has been monitored through deep sequencing of ribosome-protected mRNAs [39]. We obtain synthesis rate of Ime2 based on its protein production relative to Ime1 and synthesis rate of Ime1.

The half-life of Ime1 is 0.5 hour in the presence of Ime2 and 1 hour in the absence of Ime2 [3]. Thus, degradation rates of Ime1 in the presence and absence of Ime2 are approximated as 2/hour (ln2/0.5 hour ≈ 2/hour) and 1/hour (ln2/1 hour ≈ 1/hour), respectively, assuming that Ime1 degrades exponentially with time. Accordingly, both Ime2-independent and Ime2-activated Ime1 degradation rates are set to 1/hour. We assume the phosphorylation status does not influence Ime1 degradation; therefore, the same value is used for the degradation rate of pIme1. The half-life of Ime2 is much shorter, approximately 5 minutes [3]. Accordingly, the degradation rate of Ime2 is calculated to be 8/hour.

Although no kinetic data exist for phosphorylation, the dephosphorylation rate is estimated to be higher than the phosphorylation rate for Rim11 and Sok2 because the unphosphorylated forms of proteins are active during meiosis [2,6]. On the other hand, phosphorylated Ume6 is dominant under meiotic conditions; its rate constant for phosphorylation is higher than that of dephosphorylation [18].

### Numerical simulations

The ODE model is implemented in MATLAB. Numerical simulation of the model is performed with MATLAB using a non-stiff solver ode45. Numerical results are confirmed with other non-stiff or stiff solvers: ode23, ode113, ode15s, ode23s, and ode23t.

### Parameter sensitivity analysis

We perform global sensitivity analyses using the software package SBML-SAT [41]. A global sensitivity analysis explores the variation of model output to simultaneous perturbation of all parameters over a large range. The method of multi-parametric sensitivity analysis is utilized, which implements Latin Hypercube Sampling to randomly generate parameter values from a given range using uniform distributions. Parameter range is within one order of magnitude larger and smaller than baseline values; a total of 5,000 samplings are performed for each analysis. For each randomly generated parameter set, an objective function is computed by the sum of square errors between model outputs from random and baseline parameter sets. Each parameter set is classified as “acceptable” or “unacceptable” if the objective function value is smaller or larger than the average of all objective function values, respectively. The cumulative frequency is calculated for both acceptable and unacceptable cases. Parameter sensitivity is defined by the maximum distance of the two cumulative frequencies according to the Kolmogorov-Smirnov statistics. Therefore, sensitivity is in the range of 0 and 1; more important parameters have larger sensitivity values.

### Bifurcation analysis

We use the software XPPAUT (http://www.math.pitt.edu/~bard/xpp/xpp.html) for bifurcation analysis. Baseline parameter values and initial conditions are applied. Numerically stable and unstable steady states of the ODE model are determined as a function of a bifurcation parameter.

## Competing interests

The authors declare that they have no competing interests.

## Authors’ contributions

DR, YS and PY carried out the studies. PY conceived of the project. DR, YS and PY drafted the manuscript. All authors read and approved the final manuscript.

## Supplementary Material

Additional file 1 **InitialConditions.xls.** Randomization of initial conditions. Click here for file

Additional file 2 **Debjit031213S.pdf.** Tables S1,S2, S3, S4, S5, S6, S7 and S8, Figures S1, S2, S3, S4, S5, S6, S7, S8 and S9. Click here for file

Additional file 3 **YeastMeioticInitiation.xml.** The odel in the Systems Biology Markup Language format. Click here for file
