# Supplementary material for: Dynamic modeling of yeast meiotic initiation
Source: BMC Syst Biol. 2013 May 1;7:37. doi: 10.1186/1752-0509-7-37 (PMC3772702; doi:10.1186/1752-0509-7-37)
Supplement: Additional file 2 — Debjit031213S.pdf. Tables S1,S2, S3, S4, S5, S6, S7 and S8, Figures S1, S2, S3, S4, S5, S6, S7, S8 and S9. [file 1752-0509-7-37-S2.pdf]

Additional file 2

Dynamic modeling of yeast meiotic initiation

Debjit Ray[1,2], Yongchun Su[1], Ping Ye[1,3,§]

[1]School of Molecular Biosciences, Washington State University, PO Box 647520, Pullman, Washington 99164, USA

[2]Biological Systems Engineering, Washington State University, Pullman, Washington 99164, USA

[3]Center for Reproductive Biology, Washington State University, Pullman, Washington 99164, USA

[§]Corresponding author

Email addresses:

DR: debjit_ray@wsu.edu

YS: yongchun.su@wsu.edu

PY: pye@wsu.edu

Table S1 - The *rim11* knockout model

(2) $$\frac{dpUme6}{dt} = -u_{ume6} \cdot pUme6$$

(3) $$\frac{dpSok2}{dt} = p_{sok2} \cdot \frac{c_{sok2}}{c_{sok2} + \mathrm{Im}e1} \cdot (1 - pSok2) - u_{sok2} \cdot pSok2$$

(4) $$\frac{d\mathrm{Im}e1}{dt} = s_{ime1} \cdot \frac{c_{ime1}}{c_{ime1} + pSok2} - d_{ime1} \cdot \mathrm{Im}e1 - d'_{ime1} \cdot \mathrm{Im}e2 \cdot \frac{\mathrm{Im}e1}{c_1 + \mathrm{Im}e1}$$

(5) $$\frac{dp\mathrm{Im}e1}{dt} = -d_{pime1} \cdot p\mathrm{Im}e1$$

(6) $$\frac{d\mathrm{Im}e2}{dt} = s_{ime2} \cdot pUme6 \cdot p\mathrm{Im}e1 + s'_{ime2} \cdot \frac{\mathrm{Im}e2^5}{c_2^{\,5} + \mathrm{Im}e2^5} - d_{ime2} \cdot \frac{\mathrm{Im}e2}{c_3 + \mathrm{Im}e2}$$

Table S2 - The *ume6* knockout model

$$(1)\quad \frac{dRim11}{dt} = u_{rim11} \cdot (1 - Rim11) - p_{rim11} \cdot Rim11$$

$$(3)\quad \frac{dpSok2}{dt} = p_{sok2} \cdot \frac{c_{sok2}}{c_{sok2} + \mathrm{Ime1}} \cdot (1 - pSok2) - u_{sok2} \cdot pSok2$$

$$(4)\quad \frac{d\mathrm{Ime1}}{dt} = s_{ime1} \cdot \frac{c_{ime1}}{c_{ime1} + pSok2} - p_{ime1} \cdot Rim11 \cdot \mathrm{Ime1} - d_{ime1} \cdot \mathrm{Ime1} - d'_{ime1} \cdot \mathrm{Ime2} \cdot \frac{\mathrm{Ime1}}{c_1 + \mathrm{Ime1}}$$

$$(5)\quad \frac{dp\mathrm{Ime1}}{dt} = p_{ime1} \cdot Rim11 \cdot \mathrm{Ime1} - d_{pime1} \cdot p\mathrm{Ime1}$$

$$(6)\quad \frac{d\mathrm{Ime2}}{dt} = s'_{ime2} \cdot \frac{\mathrm{Ime2}^5}{c_2^{\,5} + \mathrm{Ime2}^5} - d_{ime2} \cdot \frac{\mathrm{Ime2}}{c_3 + \mathrm{Ime2}}$$

Table S3 - The *sok2* knockout model

(1) $$\frac{dRim11}{dt} = u_{rim11} \cdot (1 - Rim11) - p_{rim11} \cdot Rim11$$

(2) $$\frac{dpUme6}{dt} = p_{ume6} \cdot Rim11 \cdot (1 - pUme6) - u_{ume6} \cdot pUme6$$

(4) $$\frac{d\mathrm{Im}e1}{dt} = s_{ime1} - p_{ime1} \cdot Rim11 \cdot \mathrm{Im}e1 - d_{ime1} \cdot \mathrm{Im}e1 - d'_{ime1} \cdot \mathrm{Im}e2 \cdot \frac{\mathrm{Im}e1}{c_1 + \mathrm{Im}e1}$$

(5) $$\frac{dp\mathrm{Im}e1}{dt} = p_{ime1} \cdot Rim11 \cdot \mathrm{Im}e1 - d_{pime1} \cdot p\mathrm{Im}e1$$

(6) $$\frac{d\mathrm{Im}e2}{dt} = s_{ime2} \cdot pUme6 \cdot p\mathrm{Im}e1 + s'_{ime2} \cdot \frac{\mathrm{Im}e2^5}{c_2^5 + \mathrm{Im}e2^5} - d_{ime2} \cdot \frac{\mathrm{Im}e2}{c_3 + \mathrm{Im}e2}$$

Table S4 - The *ime1* knockout model

(1) $$\frac{dRim11}{dt} = u_{rim11} \cdot (1 - Rim11) - p_{rim11} \cdot Rim11$$

(2) $$\frac{dpUme6}{dt} = p_{ume6} \cdot Rim11 \cdot (1 - pUme6) - u_{ume6} \cdot pUme6$$

(3) $$\frac{dpSok2}{dt} = p_{sok2} \cdot (1 - pSok2) - u_{sok2} \cdot pSok2$$

(6) $$\frac{d\mathrm{Im}e2}{dt} = s_{ime2} \cdot \frac{\mathrm{Im}e2^5}{c_2^{\,5} + \mathrm{Im}e2^5} - d_{ime2} \cdot \frac{\mathrm{Im}e2}{c_3 + \mathrm{Im}e2}$$

Table S5 - The *ime2* knockout model

$$(1)\quad \frac{dRim11}{dt} = u_{rim11}\cdot(1-Rim11) - p_{rim11}\cdot Rim11$$

$$(2)\quad \frac{dpUme6}{dt} = p_{ume6}\cdot Rim11\cdot(1-pUme6) - u_{ume6}\cdot pUme6$$

$$(3)\quad \frac{dpSok2}{dt} = p_{sok2}\cdot \frac{c_{sok2}}{c_{sok2}+\mathrm{Ime}1}\cdot(1-pSok2) - u_{sok2}\cdot pSok2$$

$$(4)\quad \frac{d\mathrm{Ime}1}{dt} = s_{ime1}\cdot \frac{c_{ime1}}{c_{ime1}+pSok2} - p_{ime1}\cdot Rim11\cdot \mathrm{Ime}1 - d_{ime1}\cdot \mathrm{Ime}1$$

$$(5)\quad \frac{dp\mathrm{Ime}1}{dt} = p_{ime1}\cdot Rim11\cdot \mathrm{Ime}1 - d_{pime1}\cdot p\mathrm{Ime}1$$

Table S6 - The pSok2-Ime1 feedback knockout model

$$(1)\quad \frac{dRim11}{dt} = u_{rim11} \cdot (1 - Rim11) - p_{rim11} \cdot Rim11$$

$$(2)\quad \frac{dpUme6}{dt} = p_{ume6} \cdot Rim11 \cdot (1 - pUme6) - u_{ume6} \cdot pUme6$$

$$(3)\quad \frac{dpSok2}{dt} = p_{sok2} \cdot (1 - pSok2) - u_{sok2} \cdot pSok2$$

$$(4)\quad \frac{d\mathrm{Im}e1}{dt} = s_{ime1} - p_{ime1} \cdot Rim11 \cdot \mathrm{Im}e1 - d_{ime1} \cdot \mathrm{Im}e1 - d'_{ime1} \cdot \mathrm{Im}e2 \cdot \frac{\mathrm{Im}e1}{c_1 + \mathrm{Im}e1}$$

$$(5)\quad \frac{dp\mathrm{Im}e1}{dt} = p_{ime1} \cdot Rim11 \cdot \mathrm{Im}e1 - d_{pime1} \cdot p\mathrm{Im}e1$$

$$(6)\quad \frac{d\mathrm{Im}e2}{dt} = s_{ime2} \cdot pUme6 \cdot p\mathrm{Im}e1 + s'_{ime2} \cdot \frac{\mathrm{Im}e2^5}{c_2^{\,5} + \mathrm{Im}e2^5} - d_{ime2} \cdot \frac{\mathrm{Im}e2}{c_3 + \mathrm{Im}e2}$$

Table S7 - The Ime2-Ime1 feedback knockout model

$$(1)\quad \frac{dRim11}{dt} = u_{rim11} \cdot (1 - Rim11) - p_{rim11} \cdot Rim11$$

$$(2)\quad \frac{dpUme6}{dt} = p_{ume6} \cdot Rim11 \cdot (1 - pUme6) - u_{ume6} \cdot pUme6$$

$$(3)\quad \frac{dpSok2}{dt} = p_{sok2} \cdot \frac{c_{sok2}}{c_{sok2} + \mathrm{Im}e1} \cdot (1 - pSok2) - u_{sok2} \cdot pSok2$$

$$(4)\quad \frac{d\mathrm{Im}e1}{dt} = s_{ime1} \cdot \frac{c_{ime1}}{c_{ime1} + pSok2} - p_{ime1} \cdot Rim11 \cdot \mathrm{Im}e1 - d_{ime1} \cdot \mathrm{Im}e1$$

$$(5)\quad \frac{dp\mathrm{Im}e1}{dt} = p_{ime1} \cdot Rim11 \cdot \mathrm{Im}e1 - d_{pime1} \cdot p\mathrm{Im}e1$$

$$(6)\quad \frac{d\mathrm{Im}e2}{dt} = s_{ime2} \cdot pUme6 \cdot p\mathrm{Im}e1 + s'_{ime2} \cdot \frac{\mathrm{Im}e2^5}{c_2^{\,5} + \mathrm{Im}e2^5} - d_{ime2} \cdot \frac{\mathrm{Im}e2}{c_3 + \mathrm{Im}e2}$$

Table S8 - The Ime2 feedback knockout model

$$(1)\quad \frac{dRim11}{dt} = u_{rim11} \cdot (1 - Rim11) - p_{rim11} \cdot Rim11$$

$$(2)\quad \frac{dpUme6}{dt} = p_{ume6} \cdot Rim11 \cdot (1 - pUme6) - u_{ume6} \cdot pUme6$$

$$(3)\quad \frac{dpSok2}{dt} = p_{sok2} \cdot \frac{c_{sok2}}{c_{sok2} + \mathrm{Ime1}} \cdot (1 - pSok2) - u_{sok2} \cdot pSok2$$

$$(4)\quad \frac{d\mathrm{Ime1}}{dt} = s_{ime1} \cdot \frac{c_{ime1}}{c_{ime1} + pSok2} - p_{ime1} \cdot Rim11 \cdot \mathrm{Ime1} - d_{ime1} \cdot \mathrm{Ime1} - d'_{ime1} \cdot \mathrm{Ime2} \cdot \frac{\mathrm{Ime1}}{c_1 + \mathrm{Ime1}}$$

$$(5)\quad \frac{dp\mathrm{Ime1}}{dt} = p_{ime1} \cdot Rim11 \cdot \mathrm{Ime1} - d_{pime1} \cdot p\mathrm{Ime1}$$

$$(6)\quad \frac{d\mathrm{Ime2}}{dt} = s_{ime2} \cdot pUme6 \cdot p\mathrm{Ime1} - d_{ime2} \cdot \frac{\mathrm{Ime2}}{c_3 + \mathrm{Ime2}}$$

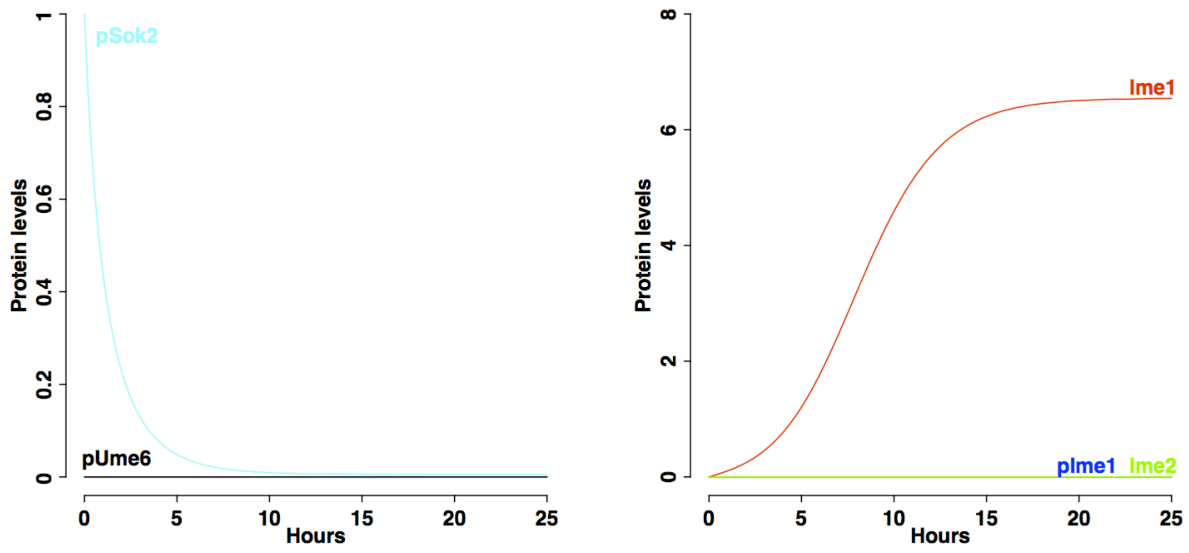

Figure S1 - Numerical simulations of the *rim11* knockout model

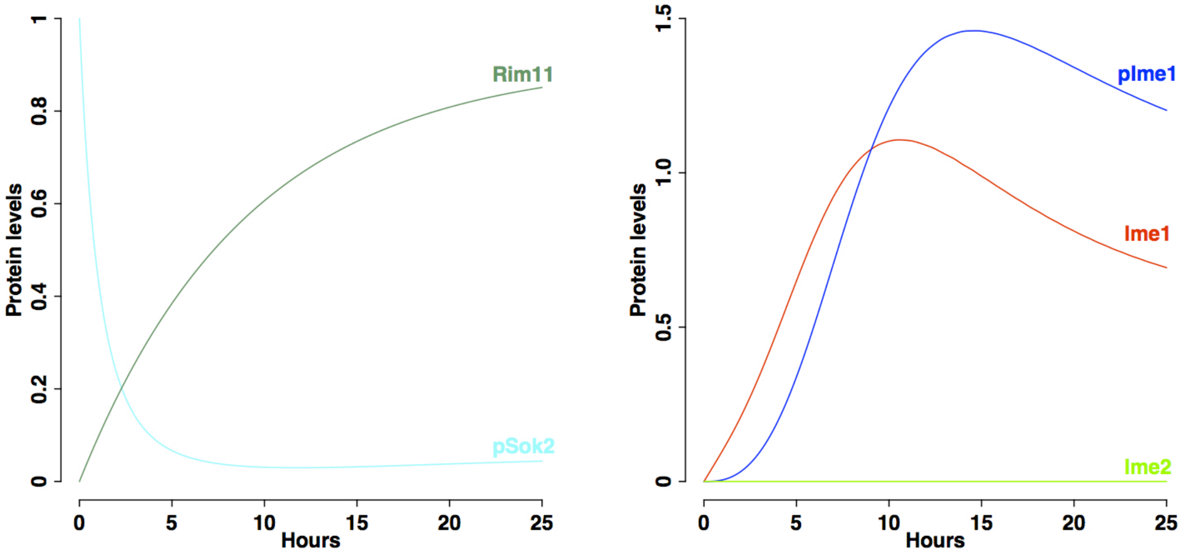

Figure S2 - Numerical simulations of the *ume6* knockout model

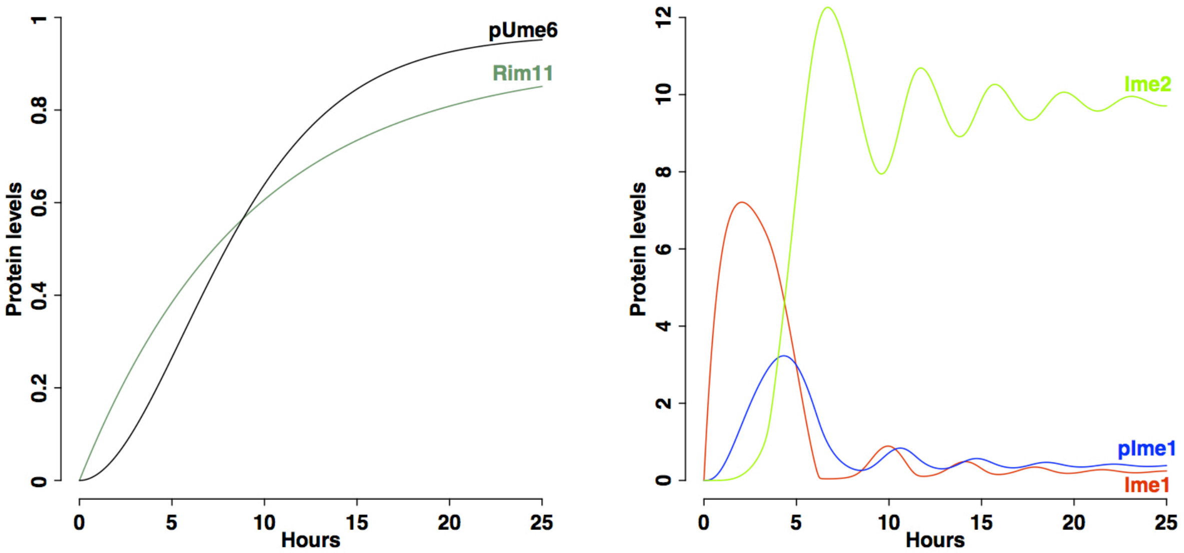

Figure S3 - Numerical simulations of the *sok2* knockout model

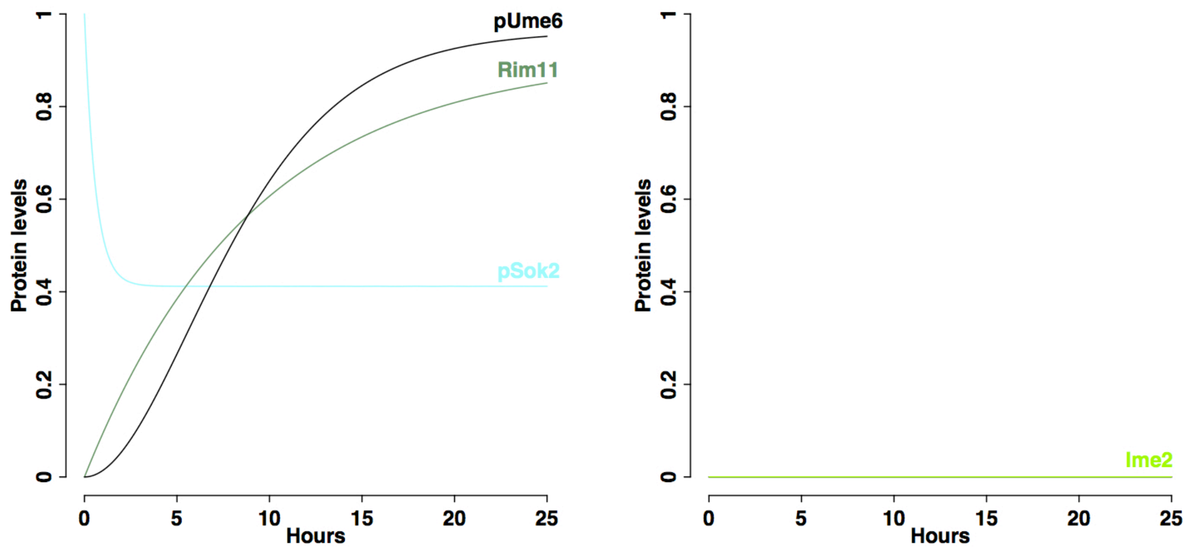

Figure S4 - Numerical simulations of the *ime1* knockout model

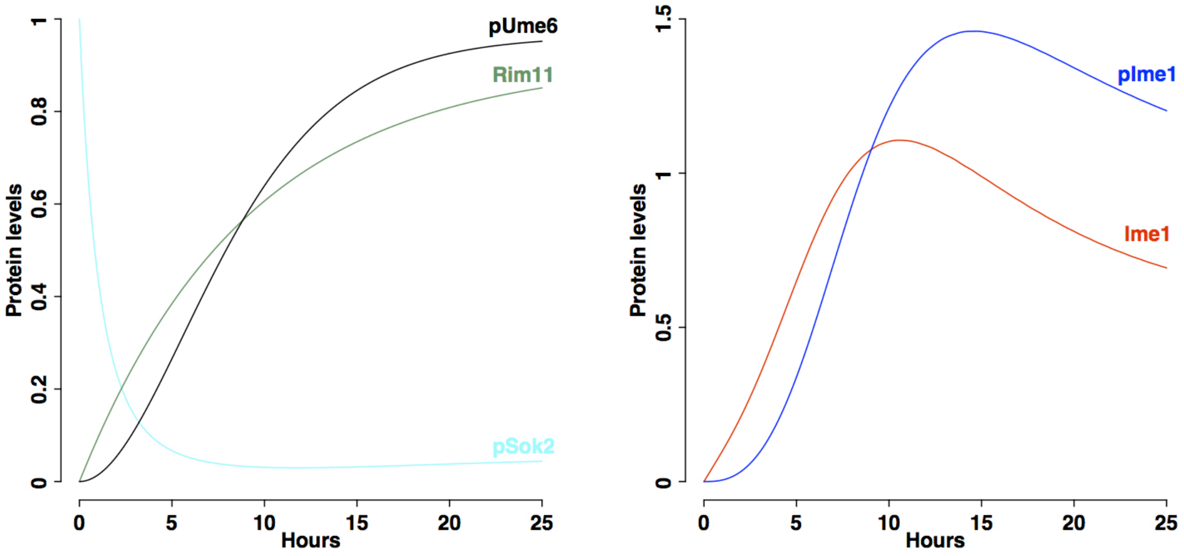

Figure S5 - Numerical simulations of the *ime2* knockout model

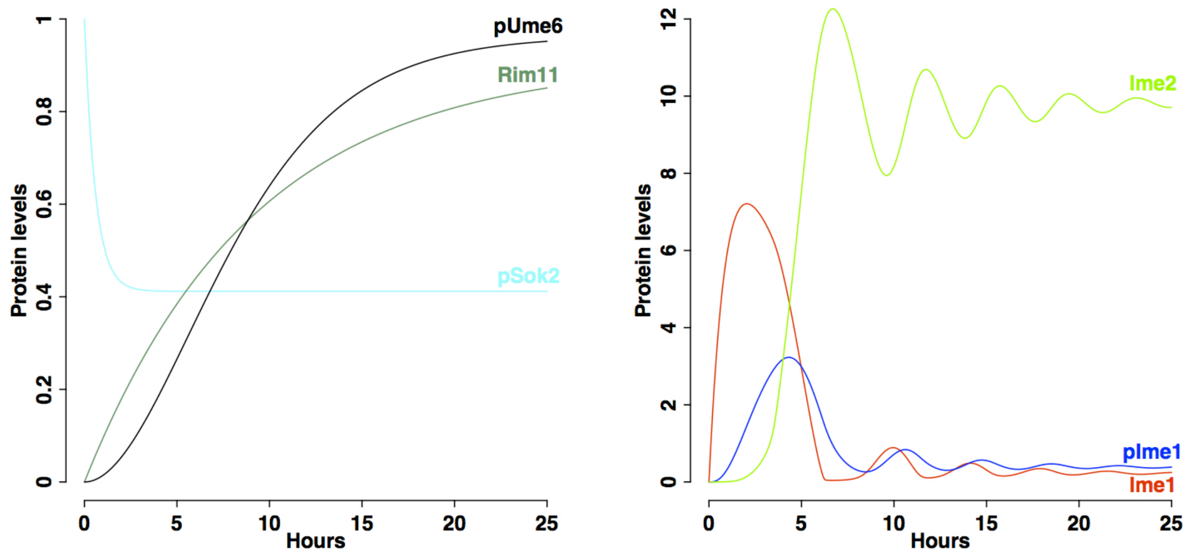

Figure S6 - Numerical simulations of the pSok2-Ime1 feedback knockout model

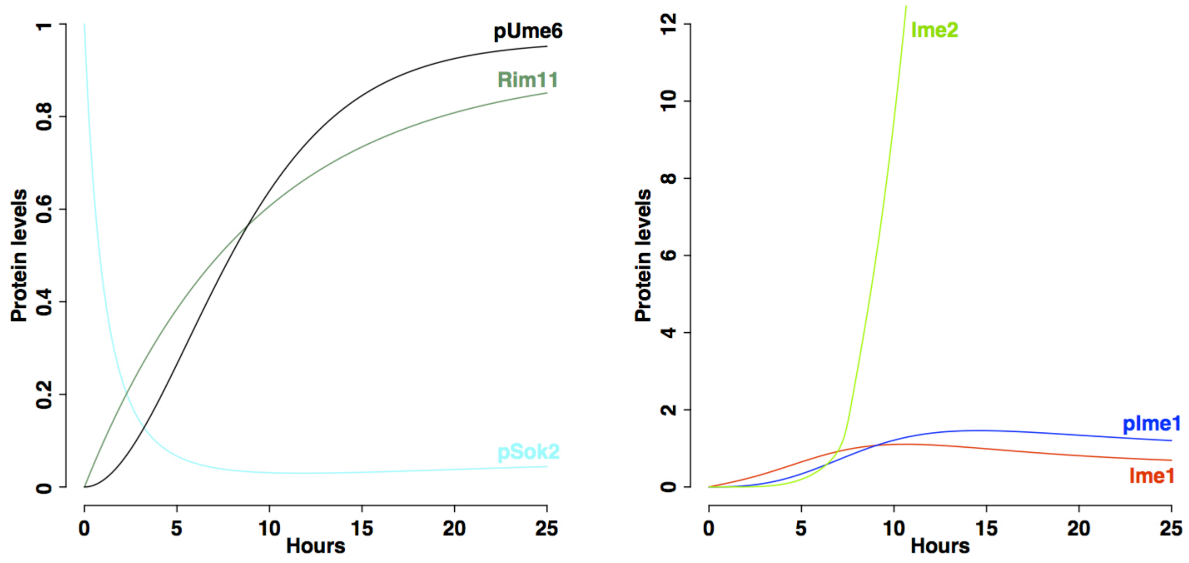

Figure S7 - Numerical simulations of the Ime2-Ime1 feedback knockout model

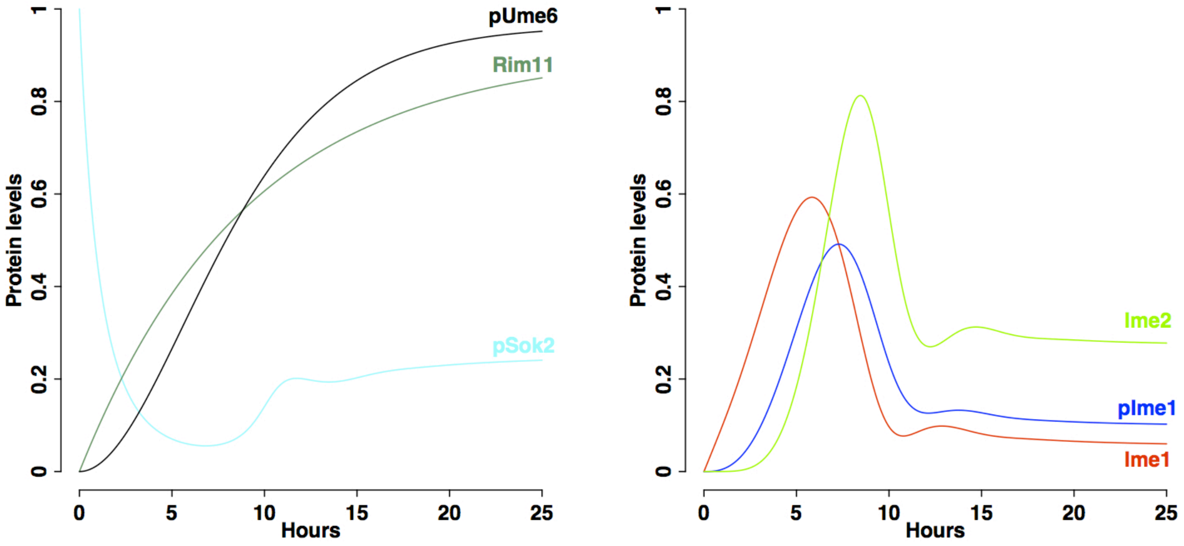

Figure S8 - Numerical simulations of the Ime2 feedback knockout model

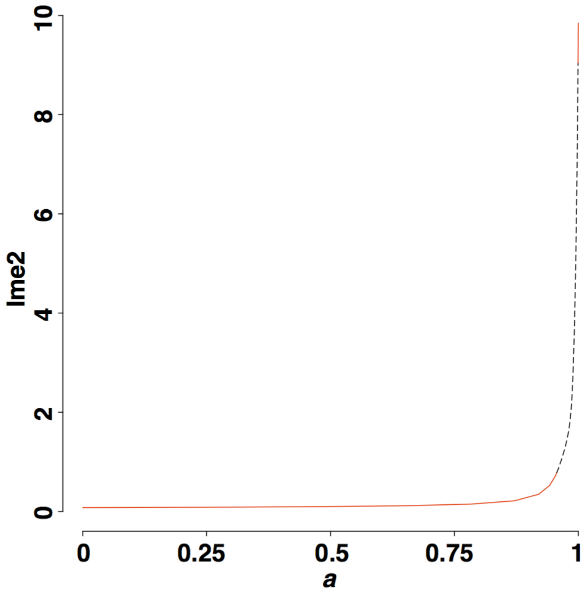

Figure S9 - Bifurcation analysis of PKA

Steady state value of Ime2 as a function of PKA. The change in PKA activity is represented by simultaneously varying p_{rim11} and p_{sok2}, phosphorylation rates of Rim11 and Sok2, respectively. This is achieved by varying a, where $p_{rim11} = 0.1 - 0.1a$ and $p_{sok2} = 7 - 7a$. Red segments represent stable steady states, whereas black segments trace unstable steady states.
